# Incorporation of NS1 and prM/M are important to confer effective protection of adenovirus-vectored Zika virus vaccine carrying E protein

**DOI:** 10.1038/s41541-018-0072-6

**Published:** 2018-07-24

**Authors:** Xinglong Liu, Linbing Qu, Xianmiao Ye, Changhua Yi, Xuehua Zheng, Mingli Hao, Wan Su, Zhipeng Yao, Peihai Chen, Shengnan Zhang, Yupeng Feng, Qian Wang, Qihong Yan, Pingchao Li, Heying Li, Feng Li, Weiqi Pan, Xuefeng Niu, Ruian Xu, Liqiang Feng, Ling Chen

**Affiliations:** 1 State Key Laboratories of Respiratory Diseases, Guangzhou Institutes of Biomedicine and Health, Chinese Academy of Sciences, Guangzhou, China; 2 School of Biomedical Sciences, Huaqiao University, Quanzhou, China; 3 University of Chinese Academy of Sciences, Beijing, China; 4 Institute of Physical Science and Information Technology, Anhui University, Hefei, China; 5 Guangzhou Eighth People’s Hospital, Guangzhou Medical University, Guangzhou, China

## Abstract

Current design of Zika virus (ZIKV) vaccine mainly considered envelope (E) as the major target antigen. Non-structural protein NS1 was seldom considered. Herein, we generated three adenovirus-vectored vaccines carrying E (Ad2-E), or premembrane/membrane (prM/M) with E (Ad2-prME), or NS1 in addition to prM/M with E (Ad2-prME-NS1). Ad2-prME induced higher neutralizing antibody response to ZIKV than Ad2-E, suggesting prM/M is important for the folding of immunogenic E. Most intriguingly, Ad2-prME-NS1 elicited the best viral inhibition when the immune sera were added to ZIKV-infected cells. In ZIKV-challenged neonatal mice born to maternally immunized dams, Ad2-prME-NS1 conferred the best protection in preventing weight loss, neurological disorders, and viral replication. Ad2-prME also conferred significant protection but was less effective than Ad2-prME-NS1, whereas Ad2-E only alleviated neurological symptoms but did not inhibit viral replication. Our study suggested that NS1 should be considered in the design of ZIKV vaccine in addition to prM/M and E.

## Introduction

Zika virus (ZIKV) is a positive-sense single-stranded RNA virus belonging to family *flaviviridae*. Since 2007, outbreaks of ZIKV infection have been continuously reported in many countries in the Americas and Southeast Asia.^1^ Historically, ZIKV infection only caused mild and self-limited symptoms.^1^ However, severe neurological diseases including congenital malformations and Guillain-Barré syndrome have been observed in recent epidemics.^2^ Sexual transmission has also been established, in addition to mosquito vectors.^1,2^ Theses unusual clinical outcomes and transmission routes have posed potential threats for global public health.^1^ No prophylactic vaccines are available.

ZIKV encodes one polyprotein which is proteolytically processed into three structural proteins: capsid (C), pre-membrane/membrane (prM/M), and envelope (E), and seven non-structural proteins ranging from NS1 to NS5. prM/M and E are both anchored in viral membrane.^3^ prM/M is cleaved into M by furin protease and facilitates post-translational folding of E.^3^ E consists of four functional domains: stem-transmembrane domain, ectodomain I (EDI), II (EDII), and III (EDIII). The N′ terminal EDI acts as a bridge between EDII and EDIII. EDII is responsible for dimerization and contains the fusion loop which mediates viral escape from lysosome. EDIII mediates viral attachment to target cells.^3^ Recently, a number of neutralizing antibodies (nAbs) have been isolated from individuals after infection with ZIKV.^4–8^ These nAbs mainly recognize EDI/II, EDIII, and tertiary or quaternary epitopes constituted by E ectodomains. EDIII-specific nAbs in general have the most potent neutralizing activities.^4–7^ So far, no NS1-specific antibodies have been reported that can inhibit ZIKV infection. Most ZIKV vaccines undergoing preclinical or clinical investigation mainly target E because it contains the major neutralizing epitopes.^2,9–19^ prM/M is also incorporated in some vaccines for assisting post-translational modification of E.^20^ Three DNA vaccines encoding prM/M and E, and one inactivated ZIKV vaccine have completed Phase I clinical trials and exhibited safety and immunogenicity.^21–23^

ZIKV NS1 has not been explored as a vaccine target antigen until very recently.^24^ It has been recognized that flaviviral NS1 has three forms: intracellular monomer, membrane-bound homodimer, and secreted homo-hexamer.^25^ Intracellular NS1 is necessary for viral replication, whereas membrane-bound and extracellular NS1 contribute to immunopathogenesis.^25^ NS1 antigenemia has been reported to be associated with the severity of human dengue disease such as dengue hemorrhagic fever.^26^ The pathogenic roles of NS1 during Dengue virus (DENV) infection include: (i) NS1 directly triggers endothelial permeability and leads to vascular leak.^27,28^ (ii) NS1 activates host immune cells including dendritic cells (DC) and macrophages and causes “cytokine storm”.^27,29,30^ (iii) NS1 binds to complement proteins and mannose-binding lectin and suppresses the lectin pathway of complement activation.^31^ (iv) Importantly, extracellular soluble NS1 can be taken up by host hepatocytes and DCs and enhances the infectivity and productivity of DENV.^32,33^ Although the roles of NS1 during ZIKV infection remain to be elucidated, one recent report showed that soluble NS1, produced by ZIKV-infected host cells, facilitates ZIKV acquisition by mosquitoes.^34^ Given that NS1 is not present on the viral particles (vp),^3^ NS1-specific antibodies are unlikely to block the attachment or entry of ZIKV to host cells. However, emerging evidence suggested that active immunization with NS1 or passive immunization with anti-NS1 antibodies protects animals from infection by DENV, West Niles virus, yellow fever virus, and Japanese encephalitis virus (JEV), possibly through inhibiting viral production as well as suppressing the pathogenic effects of NS1.^27,35–45^

Although it is believed that an effective vaccine would require E-specific antibodies to block the viral entry, we thought to evaluate if addition of NS1 into an E-based vaccine would further enhance vaccination-mediated protection. An important issue for ZIKV vaccine design should consider the most likely vaccine recipients. Since pregnant women and fetuses are the most susceptible populations, and the fetuses will suffer the most devastating consequence resulting from ZIKV infection, we aimed to design a vaccine that could confer effective protection to fetus and neonate through vertically transmitted antibodies from immunized mother. Herein, we generated three recombinant adenoviruses expressing either E (Ad2-E), or prM/M with E (Ad2-prME), or NS1 in addition to prM/M with E (Ad2-prME-NS1). A maternal immunization and neonatal challenge mouse model was established to investigate the protection efficacy of these adenovirus-vectored vaccines.

## Results

**Fig. 1:**
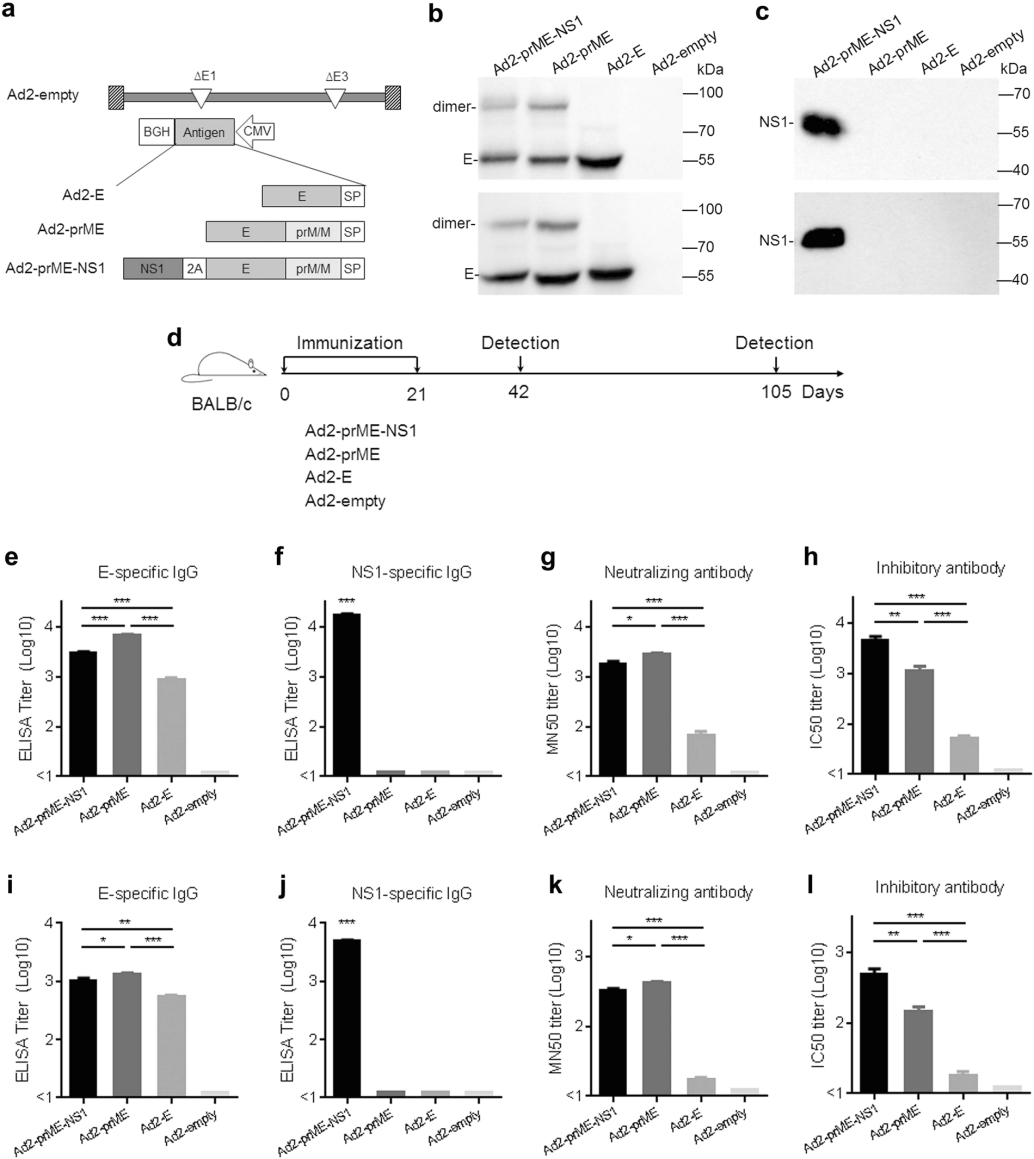
Generation of Ad2-E, Ad2-prME, and Ad2-prME-NS1 and evaluation of their immunogenicity in mice. **a** Schematic diagram of the Ad2-ZIKV vaccines in this study. SP signal peptide; 2A the coding sequence for self-cleaving 2A peptide. **b**, **c** Expression of ZIKV antigens by Vero cells infected with Ad2-E, Ad2-prME, and Ad2-prME-NS1, as well as an empty Ad2 vector (Ad2-empty) at 100 viral particles (vp) per cell. Forty-eight hours after infection, the expression of E (**b**) and NS1 (**c**) in the culture supernatants (upper panel) and cell lysates (bottom panel) were assessed by Western-blot analysis. The full, uncropped graphs can be referred to Supplementary Figs. S8–S13. **d** Timeline of immunization and immune analysis. Three or 12 weeks after the second immunization, mice were sacrificed and the serum samples were collected. **e**, **f** The binding antibodies to E (**e**) and NS1 (**f**) at 3 weeks post immunization were assessed by ELISA. The titers were calculated as the reciprocal of the sera dilution at which the optical density value at 450 nm (O.D. 450) was higher than the cut-off. **g**, **h** The neutralizing antibodies (**g**) and the inhibitory antibodies (**h**) at 3 weeks post immunization were assessed by the Fluorescence-based neutralization assay and inhibition assay, respectively. The titers were calculated as the reciprocal of the sera dilution at which the number of infected cells was reduced by 50%. **i**, **j** The binding antibodies to E (**i**) and NS1 (**j**) at 12 weeks post immunization. **k**, **l** The neutralizing antibodies (**k**) and the inhibitory antibodies (**l**) at 12 weeks post immunization. The data were representative of two independent experiments and presented as mean ± standard error (SEM). Comparison between different groups were performed by one-way analysis of variance (ANOVA, *n* = 5/group). *p < 0.05; **p < 0.01; ***p < 0.001

Three E1, E3 deleted, replication incompetent serotype 2 adenoviruses, Ad2-E, Ad2-prME, and Ad2-prME-NS1 were constructed (Fig. 1a). The genes encoding target antigens were codon-optimized for optimal expression and placed in the E1 region of adenovirus. The amino sequences of E protein, prM/M protein, and NS1 protein were from ZIKV isolate 1_0080_PF (GenBank ANO46313.1). Ad2-E, Ad2-prME, and Ad2-prME-NS1 were confirmed to mediate the expression of corresponding ZIKV antigens in infected cells (Fig. 1b, c). Dimerized E was detected in Ad2-prME or Ad2-prME-NS1-infected cells but not in Ad2-E-infected cells (Fig. 1b), suggesting that prM/M assists the formation of E dimers.^20^ NS1 was only detected in Ad2-prME-NS1-infected cells (Fig. 1c). ZIKV subviral particles (SVPs) were detected in the supernatants of cells infected with Ad2-prME-NS1 and Ad2-prME but not in those infected with Ad2-E or Ad2-empty (Supplementary Fig. S1). These results suggested that prM/M is important for the formation of SVPs, whereas the presence of NS1 in Ad2-prME-NS1 does not interfere with SVP formation.

To evaluate the immunogenicity of these vaccine candidates, 6-week-old female BALB/c mice were intramuscularly immunized twice at 1 × 10^10^ vp per mouse (Fig. 1d). Serum samples were collected at 3 and 12 weeks after the last immunization. Ad2-prME induced the highest E-binding antibody response (*p* < 0.001). E-binding antibodies elicited by Ad2-prME-NS1 were somewhat lower than that elicited by Ad2-prME (*p* < 0.001), but higher than that elicited by Ad2-E (*p* < 0.001) (Fig. 1e and Supplementary Table S1). Only Ad2-prME-NS1 immunization induced NS1-binding antibodies (Fig. 1f and Supplementary Table S1). To understand the mechanism of action of vaccine-induced antibodies, we first performed a FACS-based neutralization test (FNT). Serial dilutions of the immune sera were incubated with ZIKV for 1 h and the mixtures were added onto cultured Vero cells. Two hours later, the infectious mixtures were replaced with fresh culture media. Cells were cultured in the absence of immune sera for 4 days and then the number of ZIKV-infected Vero cells were determined by flow cytometry. Ad2-prME and Ad2-prME-NS1 showed significantly higher neutralizing titer to ZIKV than Ad2-E (*p* < 0.001) (Fig. 1g and Supplementary Table S2), suggesting that co-expression of prM/M with E is critical for inducing nAbs to ZIKV. Ad2-prME-NS1 showed slightly lower level of neutralizing activity to ZIKV as compared to Ad2-prME (*p* < 0.05). We next performed a FACS-based inhibition test (FIT) in which the immune sera were present in the culture media for 4 days and then the number of ZIKV-infected Vero cells were determined by flow cytometry. Interestingly, sera from Ad2-prME-NS1-immunized mice showed significantly higher inhibition to ZIKV than sera from Ad2-prME-immunized mice (*p* < 0.01) (Fig. 1h and Supplementary Table S2). We also tested convalescent sera collected from mice at 2 weeks after ZIKV infection using both FNT and FIT assays. The inhibitory activity of ZIKV sera was significantly higher than their neutralizing activity to ZIKV infection (*p* < 0.01) (Supplementary Fig. S3), similar to what was observed for Ad2-prME-NS1 immune sera (Supplementary Fig. S2). These results implied that NS1-specific antibodies also contribute to inhibition of ZIKV in cultured cells, most likely at the post-entry stage.

To further confirm the inhibitory effects of NS1-specific antibodies, we performed antibody depletion assays using purified NS1 and E proteins. Depletion using NS1 protein or E protein could eliminate NS1-binding antibodies or E-binding antibodies in Ad2-prME-NS1 immune sera, respectively (Supplementary Fig. S2). Depletion of E-binding antibodies significantly decreased the inhibitory effect of Ad2-prME-NS1 immune sera (*p* < 0.001). Depletion of NS1-binding antibodies also significantly lowered the inhibitory effect of Ad2-prME-NS1 immune sera (*p* < 0.001) (Supplementary Fig. S2), suggesting that NS1-specific antibodies did contribute to the suppression of ZIKV infection. Depletion of both E-binding and NS1-binding antibodies totally abolish the inhibitory effect of Ad2-prME-NS1 immune sera (Supplementary Fig. S2), further supporting the inhibitory effect of NS1-specific antibodies on ZIKV infection. We also examined the inhibitory activities of ZIKV sera before and after depletion of E-binding antibodies or NS1-binding antibodies. Similar to Ad2-prME-NS1 immune sera, depletion of E-binding or NS1-binding antibodies significantly attenuated the inhibitory activity of ZIKV antiserum (*p* < 0.001), whereas depletion of both E-binding and NS1-binding antibodies further attenuated the inhibitory activity (*p* < 0.001) (Supplementary Fig. S3). Similar results were also obtained for serum samples collected at 12 weeks after the last immunization, except that the titers of binding antibodies, nAbs, and inhibitory antibodies showed some decrease (Fig. 1i–l, and Supplementary Tables S1 and S2). Taken together, Ad2-prME-NS1 generated the most robust inhibitory antibody response to ZIKV, whereas Ad2-prME elicited less inhibitory antibody response than Ad2-prME-NS1 but much higher than Ad2-E.

To assess the vaccine-induced antibody titers in immunized dams at the time of birth, serum samples were collected from immunized dams at either 6 weeks or 15 weeks after the last immunization (Supplementary Fig. S4). The inhibitory activity of Ad2-prME-NS1 immune sera was the highest (*p* < 0.001–0.01), whereas the inhibitory activity of Ad2-E immune sera was the lowest (*p* < 0.001–0.05). There was no inhibitory activity in Ad2-empty immune sera (Supplementary Fig. S4), revealing a similar trend as the inhibitory antibody response before pregnancy (Fig. 1h, l).

**Fig. 2:**
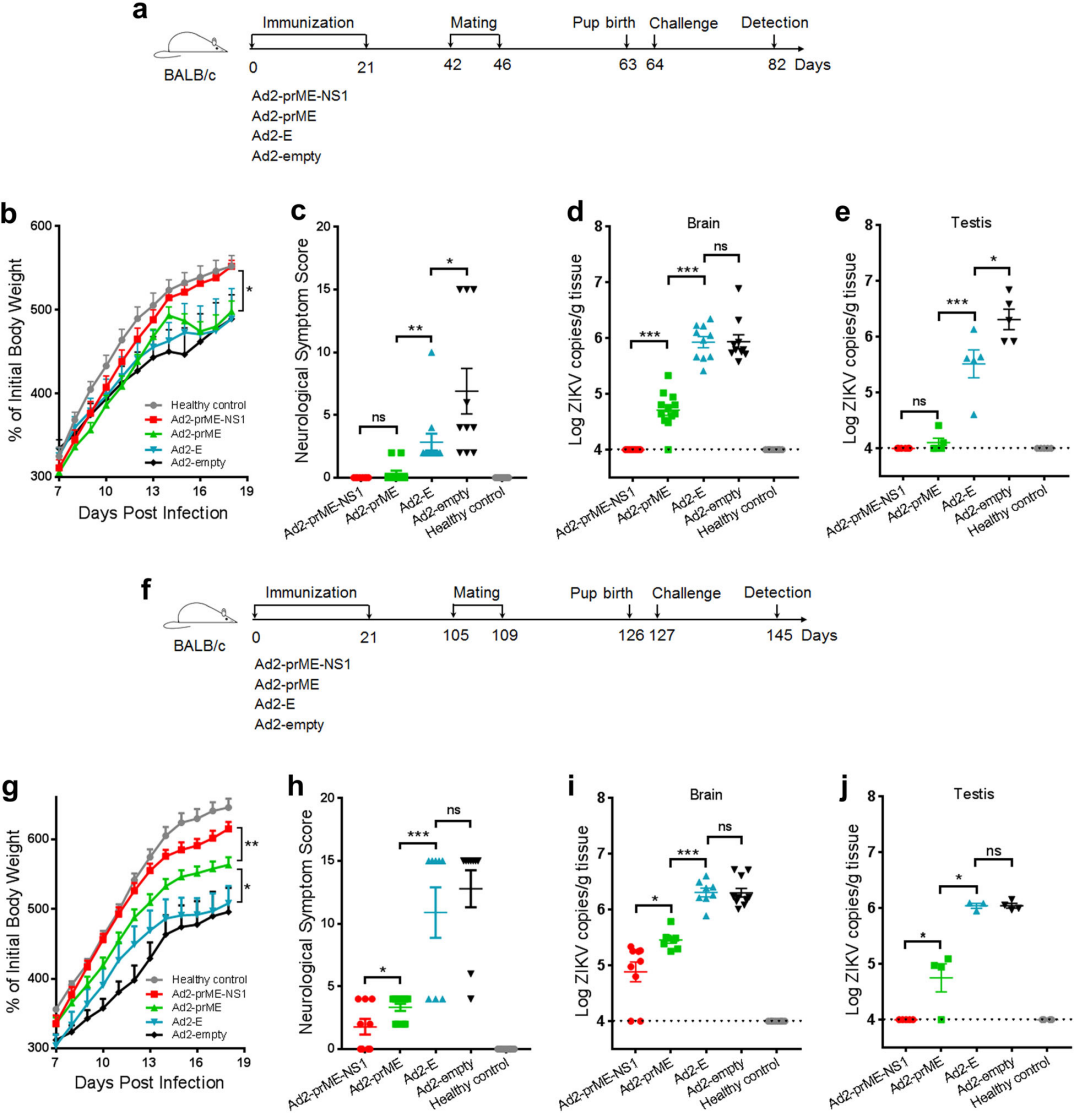
Protection efficacy against ZIKV challenge in pups born to dams immunized with Ad2-E, Ad2-prME, and Ad2-prME-NS1. **a** Schematic diagram of ZIKV challenge of pups born at 6 weeks after the completion of maternal immunization. The pups were challenged with 1.2 × 10^3^ PFU ZIKV via intraperitoneal injection at 1 day after birth. The male and female pups in each group were listed in Supplementary Table S3. Eighteen days after the challenge, pups were sacrificed. Unchallenged pups were used as healthy control. **b** The body weight of ZIKV-challenged pups. **c** The scores for neurological symptoms of ZIKV-challenged neonatal mice. The designation of neurological scores is described in Supplementary Materials and Methods. **d**, **e** The viral loads in the brain (*n* = 9–12 per group) (**d**) or testis (*n* = 5–6 per group) (**e**). Total RNA were extracted from the homogenates of the brain or testis. ZIKV genomic RNA was evaluated using one-step Q-PCR. The viral loads were expressed as the genome copy numbers per gram tissue. **f** Schematic diagram of ZIKV challenge of pups born at 15 weeks after the completion of maternal immunization. The pups were challenged as described above. Eighteen days after challenge, pups were sacrificed. Unchallenged pups were used as healthy control. **g** The body weight of ZIKV-challenged pups. **h** The scores for neurological symptoms of ZIKV-challenged pups. **i**, **j** The viral loads in the brain (*n* = 8–11 per group) (**i**) or testis (*n* = 3–5 per group) (**j**). The dotted lines indicate the limit of detection. The data were representative of two independent experiments and presented as mean ± SEM. Comparison between different groups were performed by one-way ANOVA. *p < 0.05; **p < 0.01; ***p < 0.001; ns no significance

To assess whether these vaccine candidates confer protection against ZIKV infection, the pups born at 6 weeks after the last immunization by 15-week-old maternal mice were intraperitoneally challenged with a ZIKV isolate GZ02 (GenBank KX056898.1) at 1.2 × 10^3^ plaque-forming units (PFU) per mouse at 24 h after birth (Fig. 2a). Maternal immunization with Ad2-prME-NS1 completely prevented neurological disorders and weight loss in ZIKV-infected pups (Fig. 2b, c). Ad2-prME also suppressed neurological abnormality but was less effective than Ad2-prME-NS1 in preventing weight loss (*p* < 0.05) (Fig. 2b, c). Ad2-E, however, only decreased the severity of neurological diseases (*p* < 0.05), but did not prevent weight loss (Fig. 2b, c). The brains of pups born to Ad2-prME-NS1 or Ad2-prME-immunized dams have similar weight to those of the healthy control pups (Supplementary Fig. S5a), whereas the brains of pups born to Ad2-E or Ad2-empty-immunized dams showed significantly lowered weight than healthy control pups (*p* < 0.01). This result suggested that maternal immunization with Ad2-prME-NS1 and Ad2-prME but not Ad2-E protects against ZIKV-induced microcephaly. Histological examination showed that immunization with Ad2-prME-NS1 nearly completely prevented meningeal inflammation caused by ZIKV infection (Supplementary Fig. S5c). Immunization with Ad2-prME also alleviated meningeal inflammation in the brains but was not as effective as Ad2-prME-NS1. Immunization with Ad2-E showed no obvious protection against ZIKV-induced meningeal inflammation (Supplementary Fig. S5). Quantitative PCR analysis revealed that Ad2-prME-NS1 inhibited ZIKV infection in the neonatal brain and testis to undetectable level (Fig. 2d, e). Ad2-prME significantly lowered the viral loads (*p* < 0.001), but not as effective as Ad2-prME-NS1 in inhibiting ZIKV infection (Fig. 2d, e). Ad2-E, however, only slightly lowered the viral burden in the testis (*p* < 0.05) but not in the brain (Fig. 2d, e). These results suggested that concurrent expression of prM/M with E was critical for inducing E-specific nAbs. Importantly, the addition of NS1-specific antibodies significantly enhanced the protective efficacy of ZIKV vaccine that generates E-specific nAbs.

We also measured the binding and inhibitory antibodies in the pups at 18 days after birth. Both E-specific and NS1-specific antibodies were detected in the pups born to Ad2-prME-NS1-immunized dams, whereas only E-specific antibodies were detected in the pups born to Ad2-prME or Ad2-E-immunized dams (Supplementary Fig. S6). The pups born to Ad2-prME-NS1-immunized dams had the highest inhibitory activity against ZIKV. The pups born to Ad2-prME-immunized dams showed lower inhibitory activity than Ad2-prME-NS1 (*p* < 0.01), but were higher than pups born to Ad2-E-immunized dams (*p* < 0.01). The pups born to Ad2-empty-immunized dams had no inhibitory activity (Supplementary Fig. S6).

To assess if the protective efficacy of these vaccine candidates persist after immunization in older female mice, the pups born to 24-week-old dams were challenged with ZIKV at 1.2 × 10^3^ PFU per mouse at 24 h after birth (Fig. 2f). Ad2-prME-NS1 showed the best protection in preventing neurological disorders and weight loss (Fig. 2g, h). Ad2-prME-NS1 showed the best protection than Ad2-prME and Ad2-E in preventing brain damage (*p* < 0.05) (Supplementary Fig. S5b). Ad2-prME-NS1 completely inhibited ZIKV infection in the testis and significantly lowered the viral load in the brain (*p* < 0.05) (Fig. 2i, j). Ad2-prME also prevented neurological disorders and weight loss, and suppressed viral load in the testis and brain, but was not as effective as Ad2-prME-NS1. No significant protective effects were observed for Ad2-E at this immunization dose (Fig. 2g–j). Thus, Ad2-prME-NS1 provides the best long-lasting protection than Ad2-prME. The concurrent expression of prM/M with E, and the addition of NS1 contributed to providing the best long-lasting protection against ZIKV infection.

We also examined the protective efficacy of these adenovirus-vectored vaccine candidates in adult BALB/c mice. Six-week-old female BALB/c mice were immunized twice as earlier experiments with Ad2-E, Ad2-prME, and Ad2-prME-NS1 at 1 × 10^10^ vp per mouse. At 3 weeks after the last immunization, mice were intravenously challenged with 2.4 × 10^2^ PFU ZIKV. The plasma viral load was assessed at 1 and 4 days after ZIKV challenge. Both Ad2-prME-NS1 and Ad2-prME completely prevented ZIKV-induced viremia (Supplementary Fig. S7), indicating that both Ad2-prME and Ad2-prME-NS1 could provide complete protection in adult mice, as observed in others’ studies.^12,18^ Interestingly, Ad2-E also exhibited partial suppression on plasma viral loads (*p* < 0.01–0.05). This result suggested that maternal transmitted protection to fetal or neonatal mice require more effective vaccine than protecting adult mice. A vaccine that is effective in protecting adult mice may not be sufficient to confer maternal transmitted protection in fetal or neonatal mice.

## Discussion

Several ZIKV vaccine candidates including inactivated ZIKV vaccine, live-attenuated ZIKV vaccine, chimeric ZIKV vaccine, virus-like particles, purified E protein, viral vectors, and DNA plasmids encoding ZIKV structural proteins have been explored.^10–18,46–48^ Three DNA vaccines carrying prM/M and E, as well as one inactivated ZIKV vaccine have been tested in clinical trials with reported safety and immunogenicity.^21–23^ Among these vaccine candidates, E is regarded as the major target antigen because it contains the epitopes recognized by most identified nAbs. NS1 has not been accepted as an antigen target for ZIKV vaccine; nevertheless, it has been shown to elicit protective immunity against several other flaviviruses including DENV and JEV.^27,35–40,49,50^ Our study demonstrated that incorporation of NS1 significantly enhanced the protective efficacy of ZIKV vaccine that initially designed to contain E (Figs. 1, 2). During the submission of this manuscript, Brault et al. reported that NS1 in the context of MVA vector conferred protection in an intracerebral challenge model in adult mice, but the mechanism of protection was not illustrated.^24^ In Brault’s study, it is likely that both antibody and cell-mediated immune response play roles in protecting adult mice from ZIKV infection. However, the intracerebral injection is not the natural infection route of ZIKV.^1,2^ In our study, the maternal immunization-neonatal challenge model demands antibodies to be highly efficacious, since it relies on the antibodies to be transmitted from dams to pups, while T cells cannot be transmitted from dams to pups. Our study demonstrated that incorporation of NS1 with E-based vaccine significantly enhanced the protective efficacy (Fig. 2), further supporting the addition of NS1-specific antibodies with E-specific antibodies in vertical protection of pups. Although the role of NS1 in ZIKV infection and pathogenesis remained to be elucidated, the finding that NS1-specific antibody response contributed to protection suggested it should be considered as an important component of ZIKV vaccine.

NS1 as an antigen target may have several advantages. NS1-specific antibodies function during post-entry stage (Fig. 1), and may play auxiliary roles for E-specific antibodies in inhibiting ZIKV infection (Fig. 2 and Supplementary Figs. S2, S3). NS1 is not presented in flaviviral virion, thereby NS1-specific antibodies is unlikely to cause antibody-dependent enhancement of infection.^4^ Because NS1 circulating in the blood of ZIKV-infected hosts enhances ZIKV infectivity in mosquitoes,^34^ NS1-specific antibodies may also be able to reduce ZIKV transmission by mosquitoes.^24,34^ However, a caveat of using NS1 alone as an antigen is that NS1-mediated immunity is unlikely to block the virus attachment or entry. Immunization with NS1 can induce both antibody response and cell-mediated immune responses.^24^ Because antigen-specific T cells that can destroy virus-infected cells cannot be transmitted from mother to fetuses, we speculated that a vaccine containing NS1 alone would not be effective in protecting fetuses and thus did not include NS1 alone vaccine. It is the fetus that would have the most devastating consequence with infection of ZIKV. We showed that Ad2-prME-NS1 not only protected adult mice from ZIKV infection, but protected pups and inhibit viral replication in the brain and the testis, most likely attributed to antibodies inherited from immunized dams. These merits are especially important for preventing ZIKV infection in pregnant women and fetuses, who are the major populations susceptible to ZIKV infection.

We observed that Ad2-prME-NS1 elicited relatively lower E-binding antibodies than Ad2-prME, which may be attributed to the antigenic competition between E and NS1. In fact, similar results have been reported in DENV vaccine.^37^ pCAG-prM/E, a plasmid expressing DENV prM and E, induced significantly higher titers of anti-DENV antibodies than pCAG-prM/E/NS1, a plasmid expressing prM, E, and NS1.^37^ Accordingly, the neutralizing activity of Ad2-prME immune sera was also slightly higher than Ad2-prME-NS1 immune sera (Fig. 1g, k). The inhibitory activities of Ad2-prME-NS1 immune sera, however, resulted not only from E-specific antibodies, but also from NS1-specific antibodies. Therefore, the inhibitory activity of Ad2-prME-NS1 immune sera was higher than Ad2-prME immune sera (Fig. 1h, l). Similar as the case in DENV vaccine, the inhibitory activity of pCAG-prM/E/NS1 immune sera on DENV infection was also higher than pCAG-prM/E immune sera.^37^ Taken together with the results from our depletion assay (Supplementary Figs. S2, S3), the higher inhibitory activity of Ad2-prME-NS1 immune sera can be attributed to NS1-specific inhibitory antibodies in combination with E-specific antibodies.

NS1-specific antibodies are unlikely to directly effect on viral attachment or entry because NS1 is not present on ZIKV virons. However, NS1-specific antibodies showed suppressive effects on ZIKV infection when present throughout the cell culture after addition of ZIKV into cells (Fig. 1, Supplementary Figs. S2, S3). These results were consistent to previous studies using DENV, which indicated that NS1 enhanced the infectivity of DENV, whereas NS1-specific antibodies suppressed the vascular leak caused by DENV.^27,32,33^ NS1-specific antibodies helped to alleviate ZIKV-induced neurological diseases in our study (Fig. 2). Although the exact mechanism of NS1-specific antibodies need to be elucidated in future study, we speculated that multiple pathways, including the suppression of infectivity by blocking the bio-activity of NS1, as well as the prevention against vascular leakage, may all participant in the protection of the pups.

Although it was demonstrated that circulating NS1 in an infected mammalian host can promote ZIKV infectivity and prevalence in mosquitoes,^34^ we consider that NS1 generated by immunization of Ad2-prME-NS1 would not cause any detrimental effects. Ad2-prME-NS1 is administered locally via intramuscular injection and the dosage is unlikely to increase NS1 level in the circulation. Due to the immunogenicity of adenovirus itself, the expression of NS1 mediated by Ad2-prME-NS1 would be transient after immunization, whereas NS1-specific immune responses will develop to exert the effect. NS1-specific cellular responses could facilitate the clearance of NS1-expressing cells.^24,51–53^ NS1-specific antibodies could block NS1 bioactivity and clear the circulating NS1. The presence of NS1-specific antibodies in vaccines in ZIKV pandemic region may provide additional benefit by attenuating or blocking ZIKV infectivity in mosquitoes.

Another important finding of our study was that Ad2-prME elicited more nAb response and exhibited higher protective efficacy than Ad2-E (Figs. 1, 2), revealing that ZIKV prM/M is necessary for proper post-translational folding of E. As observed for prM/M in other flaviviruses,^20^ ZIKV prM/M may also interact with E and form heterodimers, and thereby assists E to achieve a conformation that resembles what is on the virion.^20^ Indeed, we observed SVPs in infected cells only when prM/M was co-expressed with E (Supplementary Fig. S1), which may produce E protein with immunogenicity more similar to that of the virion.^11,14,54,55^ Therefore, the use of prM/M is important for E protein to induce effective immunity and should be included in ZIKV vaccine design that requires viral vectors or non-viral vectors-mediated expression.

Recently, several ZIKV vaccine candidates based on recombinant chimpanzee adenovirus (AdC7), rhesus adenovirus (RhAd52), as well as human adenovirus (Ad5) have been evaluated in mouse and rhesus monkey models.^12,13,18^ These candidates, expressing either the ectodomain of E or prM and E, could induce protective nAb response and T cell response. Adoptive transfer studies further demonstrated the protective effects of vaccine-induced antibody response.^12,18^ Notably, we used lower vaccine dosage (1 × 10^10^ vp per mouse) than that reported by others (1 × 10^11^ vp per mouse in Abbink’s study and 4 × 10^10^ vp to 1.6 × 10^11^ vp per mouse in Xu’s study).^12,18^ Ad2-prME-NS1 demonstrated the best efficacy than other vaccine candidates in protecting neonatal mice from ZIKV infection. Adenovirus-vectored vaccines have demonstrated good safety profiles. Several hundreds of clinical trials for vaccines or gene therapies have used recombinant adenovirus as vectors without severe adverse events. Importantly, adenovirus-vectored vaccines have shown great immunogenicity in both preclinical and clinical trials. High level of antibody and cell-mediated immune responses were observed for adenovirus-vectored vaccines. Adenovirus-vectored ZIKV vaccine candidates should be considered for further evaluation in human trials if possible.

In summary, we evaluated three ZIKV vaccine candidates in the context of Ad2 vectors expressing potential ZIKV antigens using a maternal-neonatal mouse model. We demonstrated that incorporation of NS1 and prM/M are critical for conferring the best protective efficacy of adenovirus-vectored ZIKV vaccine carrying E protein. This study provided insightful information for the design of an effective ZIKV vaccine.

## Methods

Six-week-old male and female BALB/c mice were purchased from Beijing Vital River Laboratory Animal Technology Co. Ltd. All animals were bred and housed in the Animal Experimental Center of Guangzhou Institutes of Biomedicine and Health (GIBH), Chinese Academy of Sciences (CAS), according to the guidelines set by the Association for the Assessment and Accreditation of Laboratory Animal Care. The experimental protocols were approved by the Institutional Animal Care and Use Committee of GIBH (IACUC #2015014). All infectious work was conducted under biosafety level 2 conditions.

The establishment of maternal immunization and neonatal challenge model was as the following. Six-week-old female mice were immunized with Ad2-prME-NS1, Ad2-prME, and Ad2-E at 1 × 10^10^ vp per mouse in 100 μl phosphate buffer saline (PBS) through intramuscular injection. Mice injected with Ad2-empty were used as negative controls. At 3 weeks after the first immunization, mice were boosted with the same doses of the respective vaccine candidates. At 3 weeks or 12 weeks after the second immunization, the mice were sacrificed and the serum samples were collected and subjected to immunological analysis.

To evaluate the protective efficacy of Ad2-prME-NS1, Ad2-prME, and Ad2-E in neonatal mice, 6-week-old female mice were immunized similarly as mentioned above. At 3 weeks or 12 weeks after the second immunization, immunized female mice were mated with male mice for 5 days. One day after birth, the pups were challenged with ZIKV (isolate GZ02, GenBank KX056898.1) via intraperitoneal route with 1.2 × 10^3^ PFU per mouse in 20 μl PBS. Unchallenged pups were used as healthy controls. The body weight was monitored and recorded. The pups of which the body weight was lower than 70% of that of healthy control pups were designated as death. We conducted an experiment earlier in which pups were infected with ZIKV at 1, 3, or 5 days after birth, respectively. Pups challenged at 1 day after birth developed the most severe neurological symptoms as compared to pups challenged at 3 or 5 days after birth. We thus chose 24 h after birth for challenge. We also established that at a challenge dosage of 1.2 × 10^3^ PFU per mouse, 100% pups developed neurological diseases, with about 50% pups succumbed to ZIKV infection. Fifteen days after challenge, the neurological symptoms of the pups were scored in a blinded manner as described in Supplementary Materials and Methods. Finally, the pups were sacrificed and the sera, brain, and testis were harvested and subjected to histological, virological, and immunological analysis.

The generation and characterization of Ad2-prME-NS1, Ad2-prME, and Ad2-E vaccines and the methods of virological and immunological assays can be found in Supplementary Materials and Methods. All blots were processed in parallel and derived from the same experiment.

### Data availability

All data generated or analyzed during this study are included in this published article.

## Electronic supplementary material

Supplementary Information
