# Supplementary material for: Incorporation of NS1 and prM/M are important to confer effective protection of adenovirus-vectored Zika virus vaccine carrying E protein
Source: NPJ Vaccines. 2018 Jul 24;3:29. doi: 10.1038/s41541-018-0072-6 (PMC6057874; doi:10.1038/s41541-018-0072-6)
Supplement: Supplementary file 1 — Supplementary Information [file 41541_2018_72_MOESM1_ESM.pdf]

Supplementary Information

Supplementary Figures

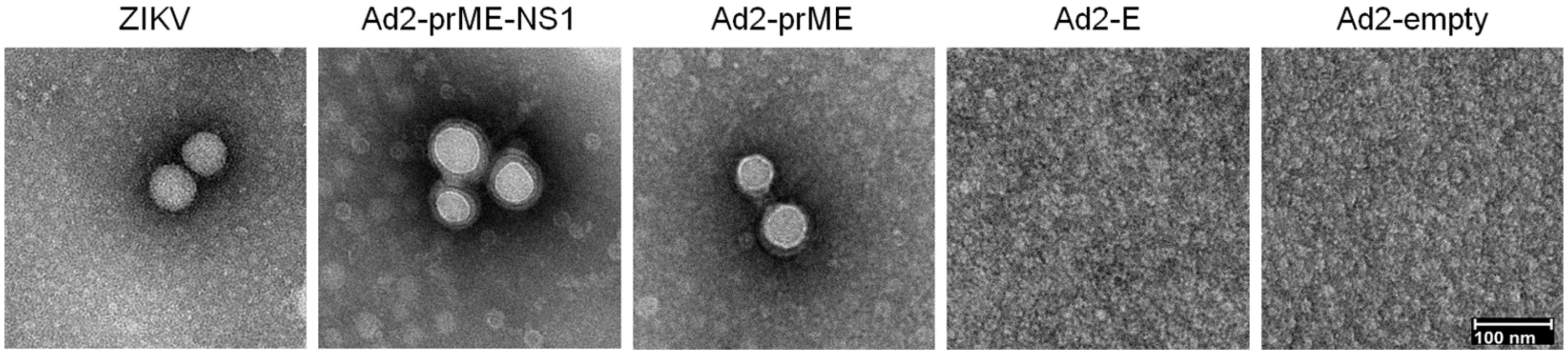

Supplementary Figure S1. Subviral particles are formed when prM/M and E are expressed concurrently. Vero cells were infected with Ad2-prME-NS1, Ad2-prME, Ad2-E and Ad2-empty. At 48 hours post infection, the culture media were collected and examined with Negative-stain electron microscopy. The ZIKV preparation used to infect cells was also examined as a control. One representative graph from each sample was shown.

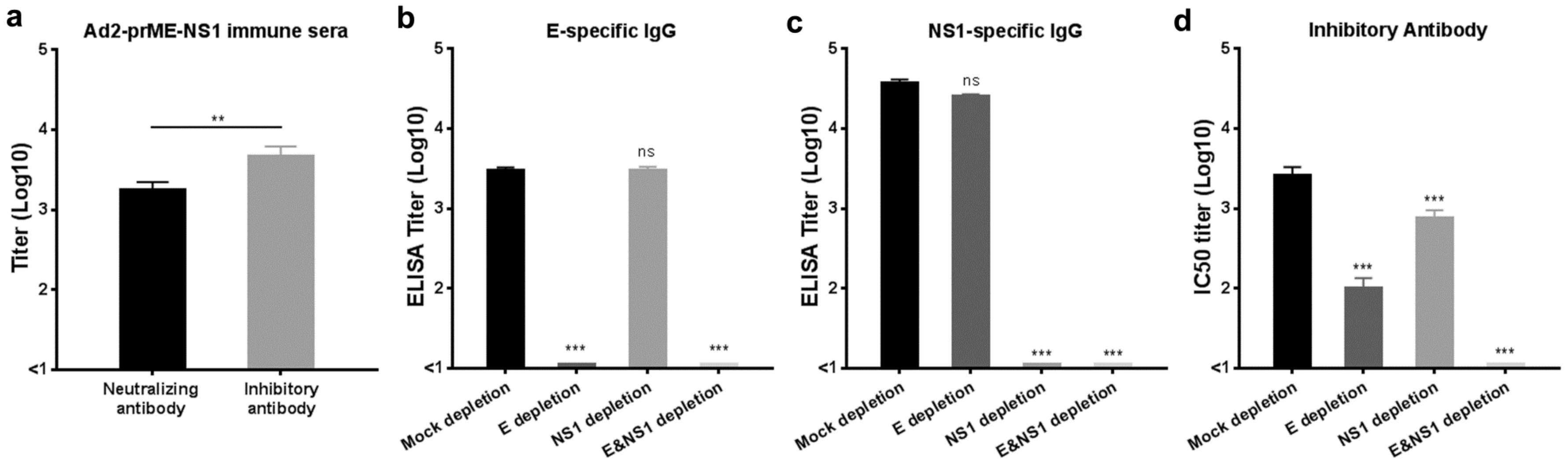

Supplementary Figure S2. The neutralizing antibody and inhibitory antibody titers of Ad2-prME-NS1 immune sera before and after specific antibody depletion. Ad2-prME-NS1 immune sera were collected at 3 weeks after the second immunization. (**a**) The titers of neutralizing antibodies and inhibitory antibodies were examined by FACS-based neutralization test (FNT) and FACS-based inhibition test (FIT). The titers were calculated as the reciprocal of the sera dilution at which the number of infected cells was reduced by 50%. (**b**) E-specific IgG antibodies in the sera before and after depletion were assessed using ELISA. (**c**) NS1-specific IgG antibodies in the sera before and after depletion were

assessed using ELISA. The titers were calculated as the reciprocal of the sera dilution at which the optical density value at 450 nm (O.D.450) was higher than the cutoff value. (**d**) The inhibitory effects of Ad2-prME-NS1 immune sera after specific antibody depletion were assessed by FIT, in which the immune sera were present in the culture media for 4 days. The data were representative of two independent experiments and presented as mean ± SEM, n = 5 per group. Comparisons were performed between sera depleted with different proteins and those depleted with PBS by Student's t-test. **, $p<0.01$; ***, $p<0.001$; ns, no significance.

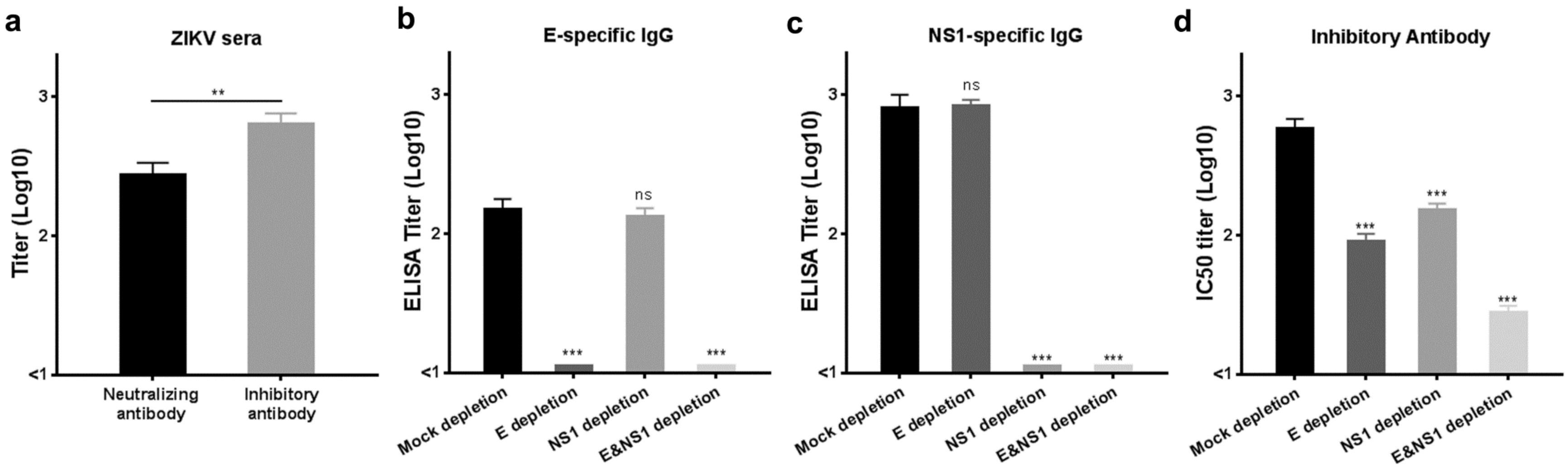

Supplementary Figure S3. The neutralizing antibody and inhibitory antibody titers of ZIKV sera before and after specific antibody depletion. ZIKV sera were collected from convalescent BALB/c mice at 2 weeks after ZIKV infection. (**a**) The titers of neutralizing antibodies and inhibitory antibodies were examined by FNT and FIT. The titers were calculated as the reciprocal of the sera dilution at which the number of infected cells was reduced by 50%. (**b**) E-specific IgG antibodies in the sera before and after depletion were assessed using ELISA. (**c**) NS1-specific IgG antibodies in the sera before and after depletion were assessed using ELISA. The titers were calculated as the reciprocal of the sera dilution at which the optical density value at 450 nm (O.D.450) was higher than the cutoff value. (**d**) The inhibitory effects of ZIKV

sera after specific antibody depletion were assessed by FIT, in which the sera were present in the culture media for 4 days. The data were representative of two independent experiments and presented as mean ± SEM, n = 5 per group. Comparisons were performed between sera depleted with different proteins and those depleted with PBS by Student's t-test. **, $p<0.01$; ***, $p<0.001$; ns, no significance.

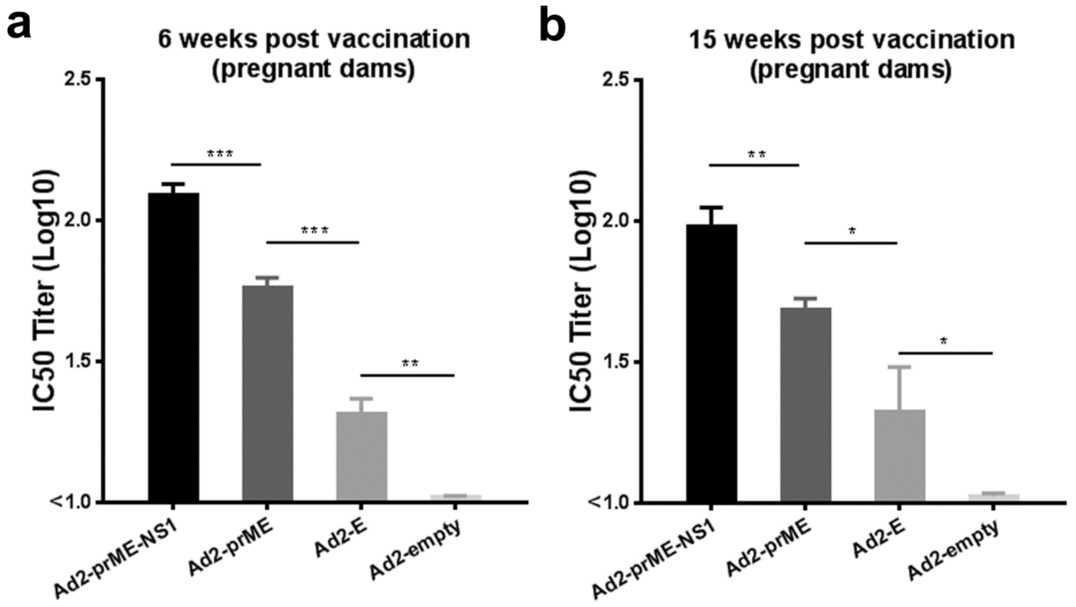

Supplementary Figure S4. The titers of inhibitory antibodies of pregnant dams at 6 and 15 weeks post immunization. The serum samples of pregnant dams were collected at one day after the birth of pups. The inhibitory antibodies were assessed by FIT. (**a**) The inhibitory antibody titers at 6 weeks post the last immunization. (**b**) The inhibitory antibody titers at 15 weeks post the last immunization. The titers were calculated as the reciprocal of the sera dilution at which the number of infected cells was reduced by 50% and expressed as IC50 titer. The data were representative of two independent experiments and presented as mean ± SEM, n=5 per group. Comparison between different groups were performed by one-way ANOVA. *, $p<0.05$; **, $p<0.01$; ***, $p<0.001$.

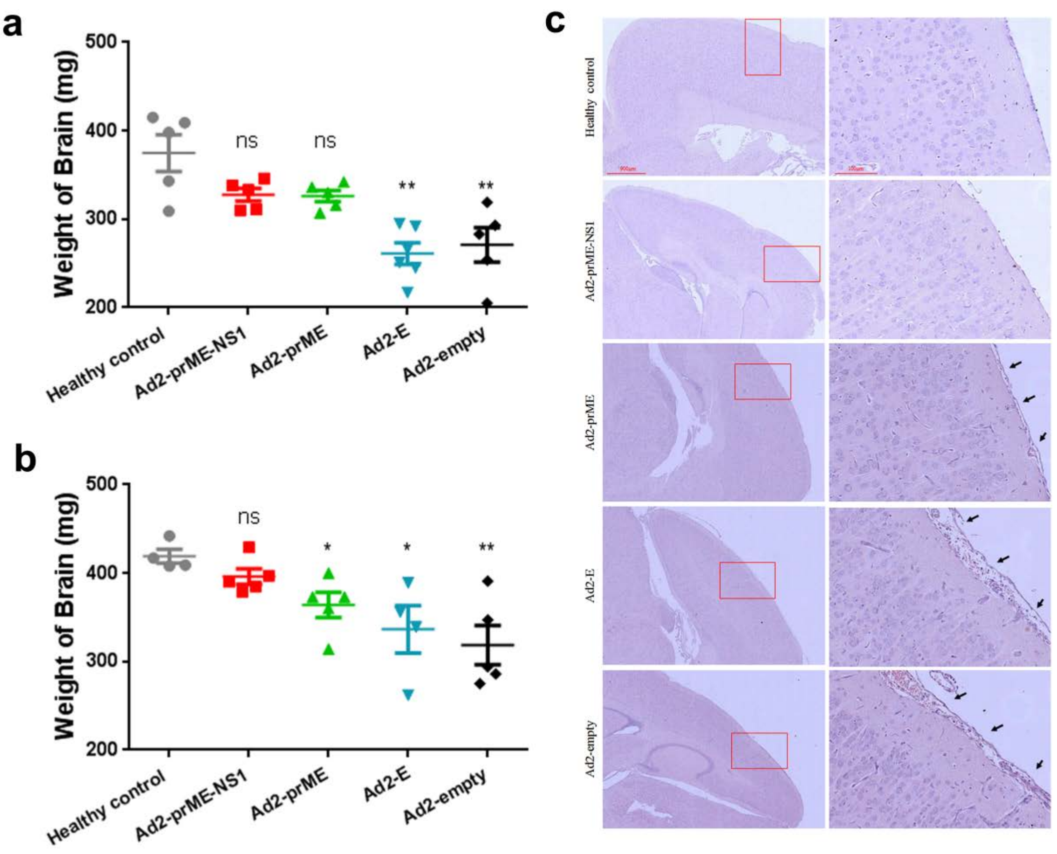

Supplementary Figure S5. Maternal immunization with Ad2-prME-NS1 prevented microcephaly and brain injury in pups. The neonatal brains from pups born at 6 weeks or 15 weeks post immunization were separated at 18 days after ZIKV challenge. Unchallenged pups born at the same time points were used as healthy controls. (**a, b**) The weight of brains from pups born at 6 weeks (**a**) or 15 weeks (**b**) post immunization. (**c**) The brain tissue sections from each group of pups born at 6 weeks post immunization were examined with H&E staining. All mice were from experiments described in Figure 2. One representative graph from each group of animals was shown. The arrows marked the inflammatory cell infiltration in the meninges. The

data were representative of two independent experiments and presented as mean ± SEM, n=4-6 per group. Comparison between ZIKV challenged pups and healthy control pups were performed by Student’s t-test. *, $p<0.05$; **, $p<0.01$; ns, no significance.

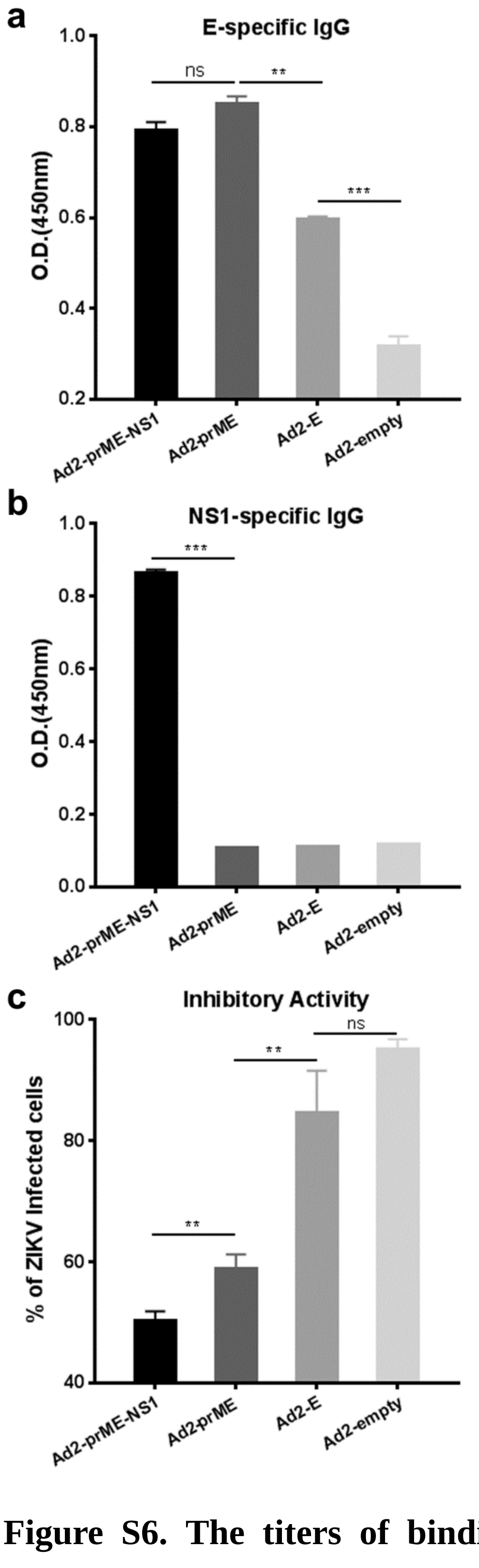

Supplementary Figure S6. The titers of binding antibodies and inhibitory antibodies in the sera of pups. At 18 days after birth, the pups born to dams immunized with Ad2 vectored ZIKV vaccines were

sacrificed and the serum samples were collected and subjected to ELISA and FIT assays. (**a**) The titers of E-specific IgG antibodies in the pups' sera were assessed with ELISA at a dilution of 1:20. Shown are the O.D.450 values. (**b**) The titers of NS1-specific IgG antibodies in the pups' sera were assessed with ELISA at a dilution of 1:20. Shown are the O.D.450 values. (**c**) The inhibitory activities of the pups' sera were assessed using FIT assay. The percentages of ZIKV-infected cells in the presence of pups' sera at a dilution of 1:20 were shown. The data were representative of two independent experiments and presented as mean ± SEM, n = 4 per group. Comparison between different groups were performed by one-way ANOVA. **, $p<0.01$; ***, $p<0.001$; ns, no significance.

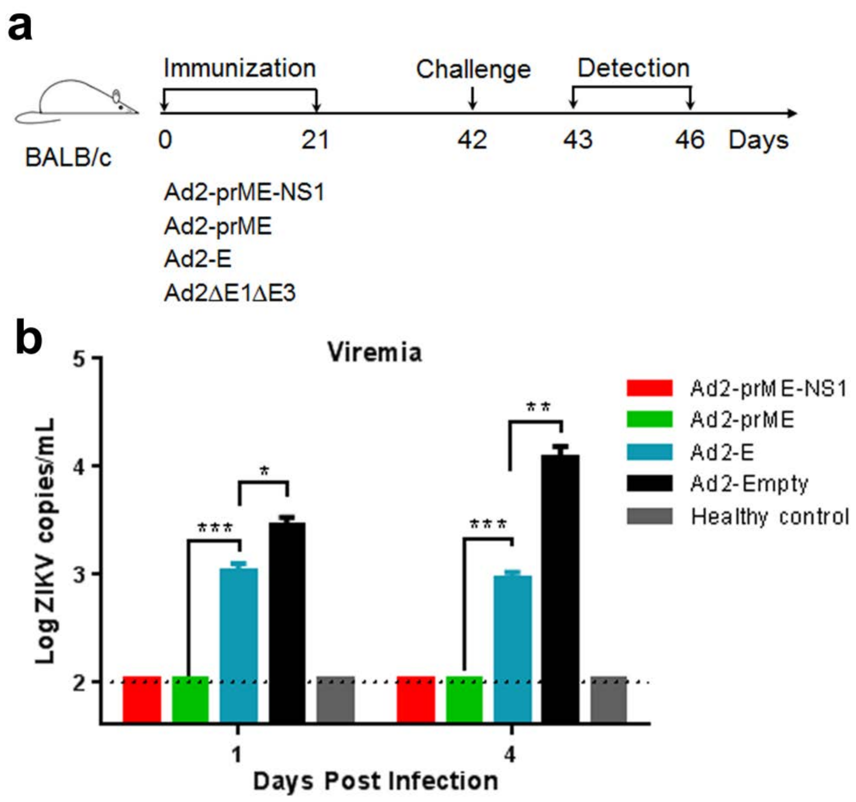

Supplementary Figure S7. The protective efficacy of Ad2 vectored ZIKV vaccines in adult mice. 6-week-old female BALB/c mice were intramuscularly immunized with 1×10^{10} vp Ad2-prME-NS1, Ad2-prME, Ad2-E and Ad2-empty twice at a three-week interval. Three weeks after the final immunization, mice were intravascularly challenged with 2.4×10^{2} PFU ZIKV. The viral loads in the serum samples were examined at 1 and 4 days post challenge. (**a**) Schematic diagram of immunization and ZIKV challenge in adult mice. (**b**) The viral loads in the plasma were assessed with Q-PCR and calculated as genome copies per milliliter serum samples. The dotted lines indicate the limit of detection. The data were from one experiment and representative of two independent experiments and presented as mean ± SEM, n = 5 per group. Comparison

between different groups were performed by one-way ANOVA. *, $p<0.05$;**, $p<0.01$; ***, $p<0.001$.

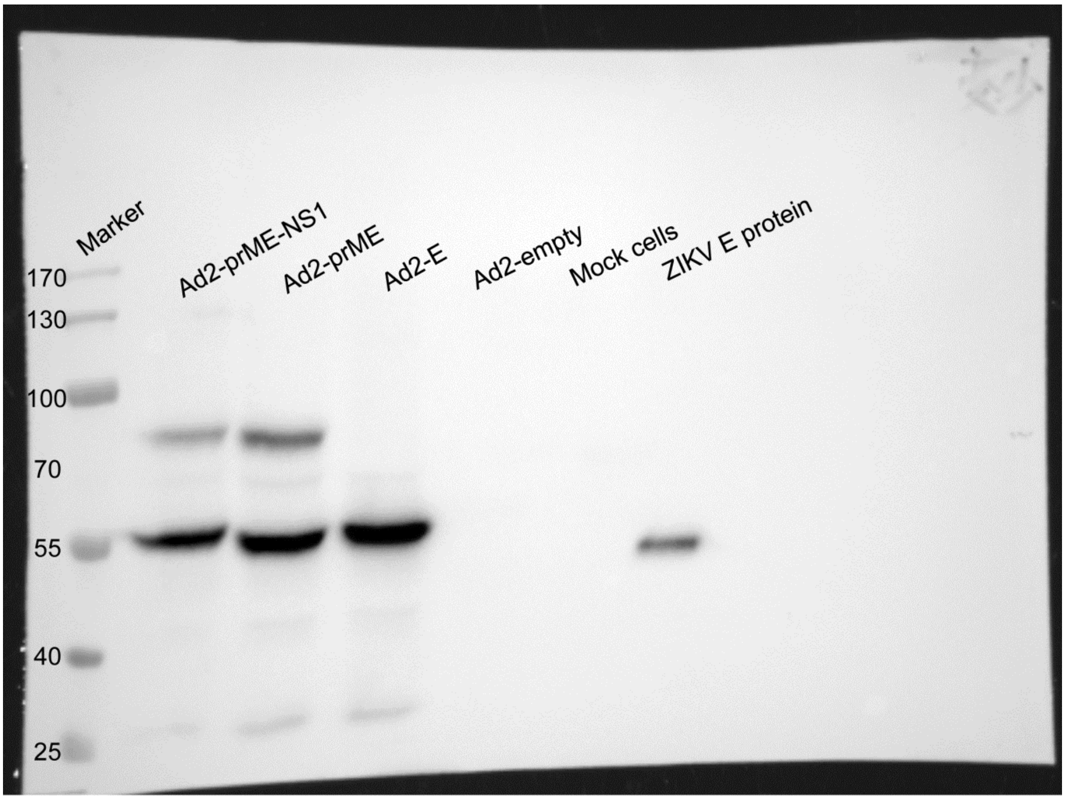

Supplementary Figure S8. Ad2 vectored vaccines mediated expression of E proteins in infected cells. The lysates of Vero cells infected with Ad2-prME-NS1, Ad2-prME, Ad2-E and Ad2-empty were assessed using Western-blot analysis with anti-E antibody. Purified ZIKV E proteins and mock infected cells and were used as positive and negative controls, respectively. The graph was taken with ChemiDoc MP Imaging System and shown.

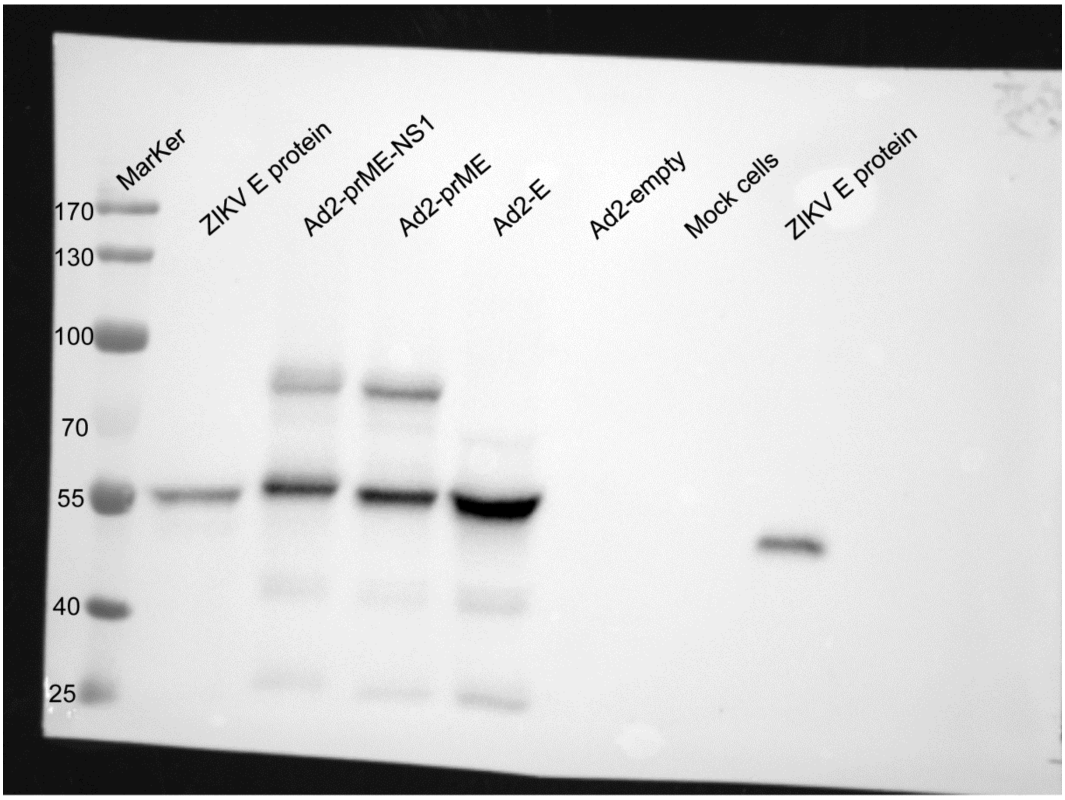

Supplementary Figure S9. Ad2 vectored vaccines mediated expression of E proteins in the culture medium of infected cells. The culture supernatants of Vero cells infected with Ad2-prME-NS1, Ad2-prME, Ad2-E and Ad2-empty were assessed using Western-blot analysis with anti-E antibody. Purified ZIKV E proteins and mock infected cells and were used as positive and negative controls, respectively. The graph was taken with ChemiDoc MP Imaging System and shown.

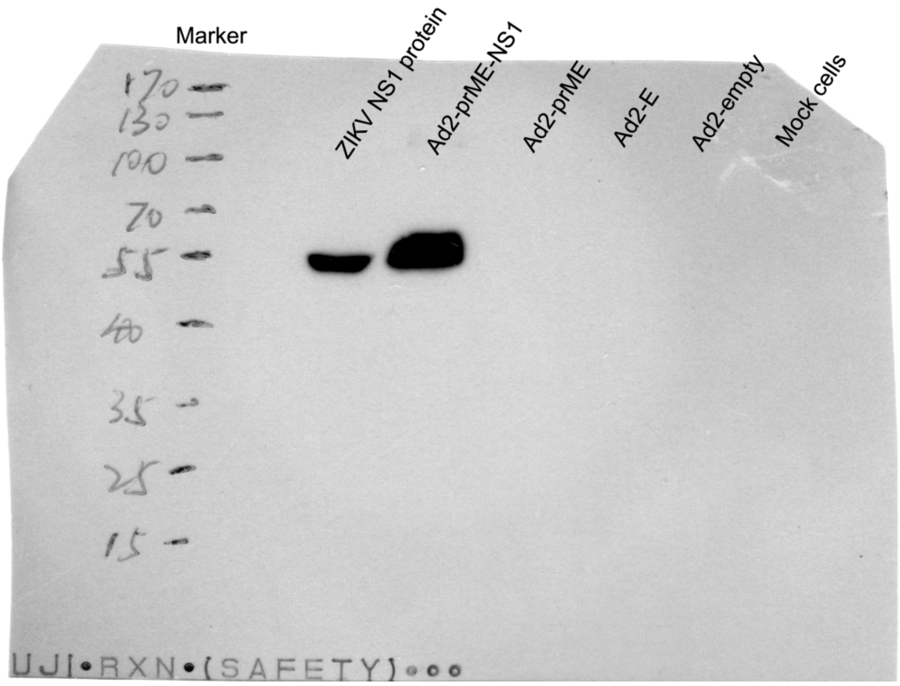

Supplementary Figure S10. Ad2-prME-NS1 mediated expression of NS1 proteins in infected cells. The lysates of Vero cells infected with Ad2-prME-NS1, Ad2-prME, Ad2-E and Ad2-empty were assessed using Western-blot analysis with anti-NS1 antibody. ZIKV infected cells and purified NS1 proteins were used as positive controls, and mock infected cells were used as negative controls. The graph was taken by scanning the developed film and shown.

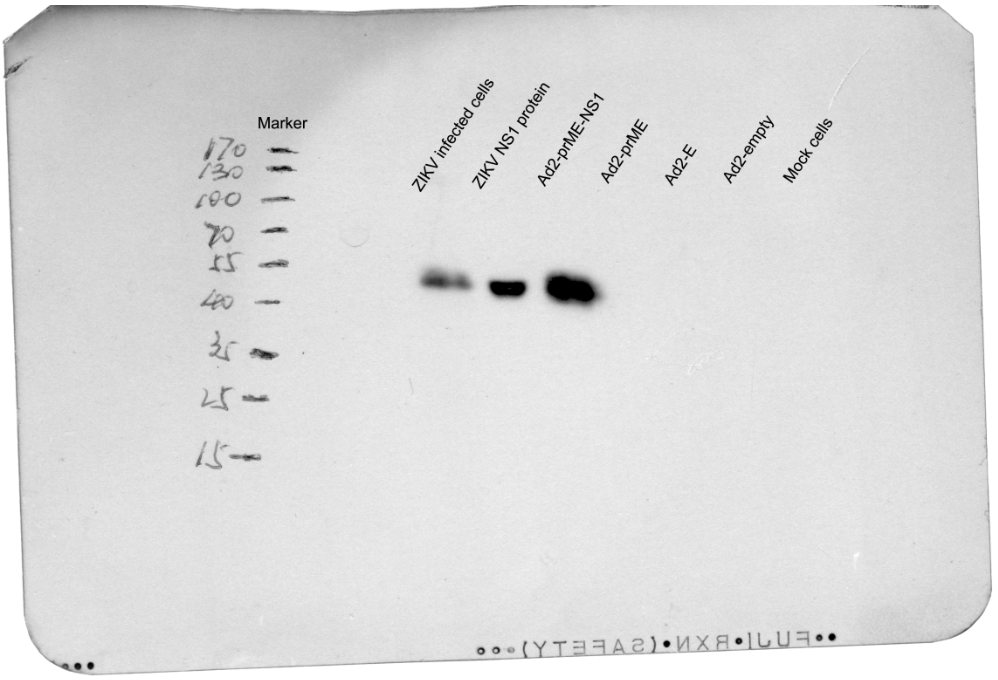

Supplementary Figure S11. NS1 proteins expressed by Ad2-prME-NS1 are secreted to culture mediums. The culture supernatants of Vero cells infected with Ad2-prME-NS1, Ad2-prME, Ad2-E and Ad2-empty were assessed using Western-blot analysis with anti-NS1 antibody. ZIKV infected cells and purified NS1 proteins were used as positive controls, and mock infected cells were used as negative controls. The graph was taken by scanning the developed film and shown.

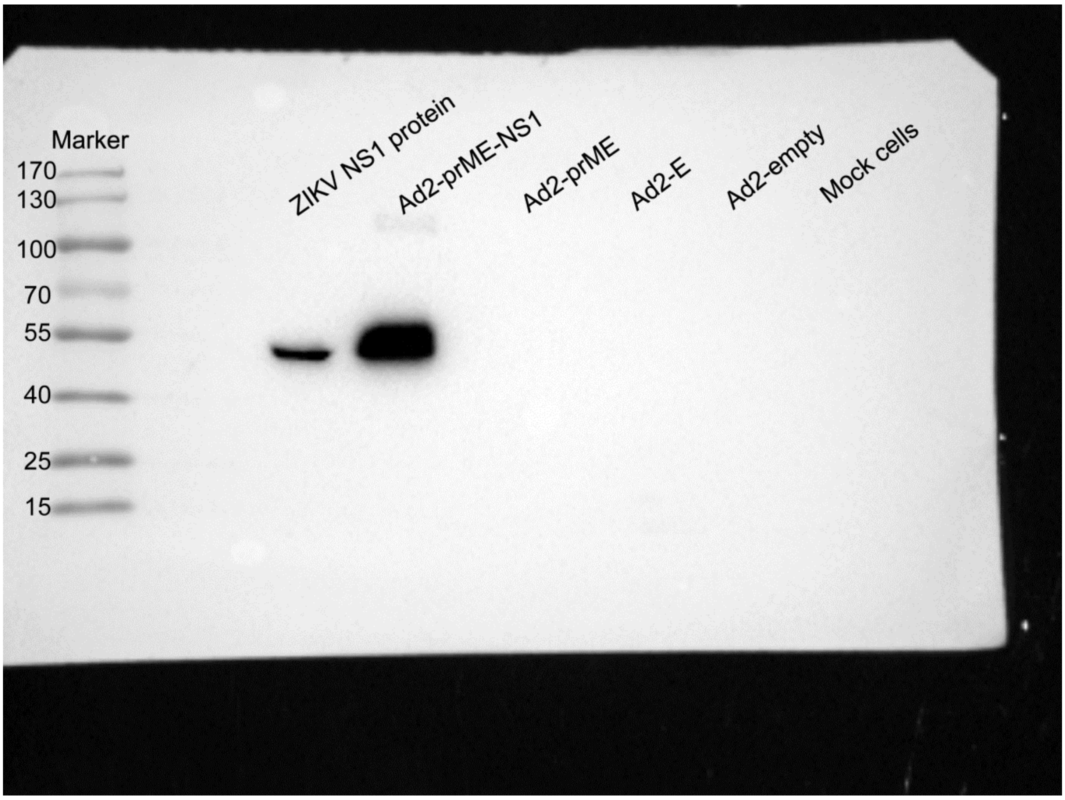

Supplementary Figure S12. Ad2-prME-NS1 mediated expression of NS1 proteins in infected cells. The lysates of Vero cells infected with Ad2-prME-NS1, Ad2-prME, Ad2-E and Ad2-empty were assessed using Western-blot analysis with anti-NS1 antibody. Purified NS1 proteins were used as positive controls, and mock infected cells were used as negative controls. The graph was taken with ChemiDoc MP Imaging System and shown.

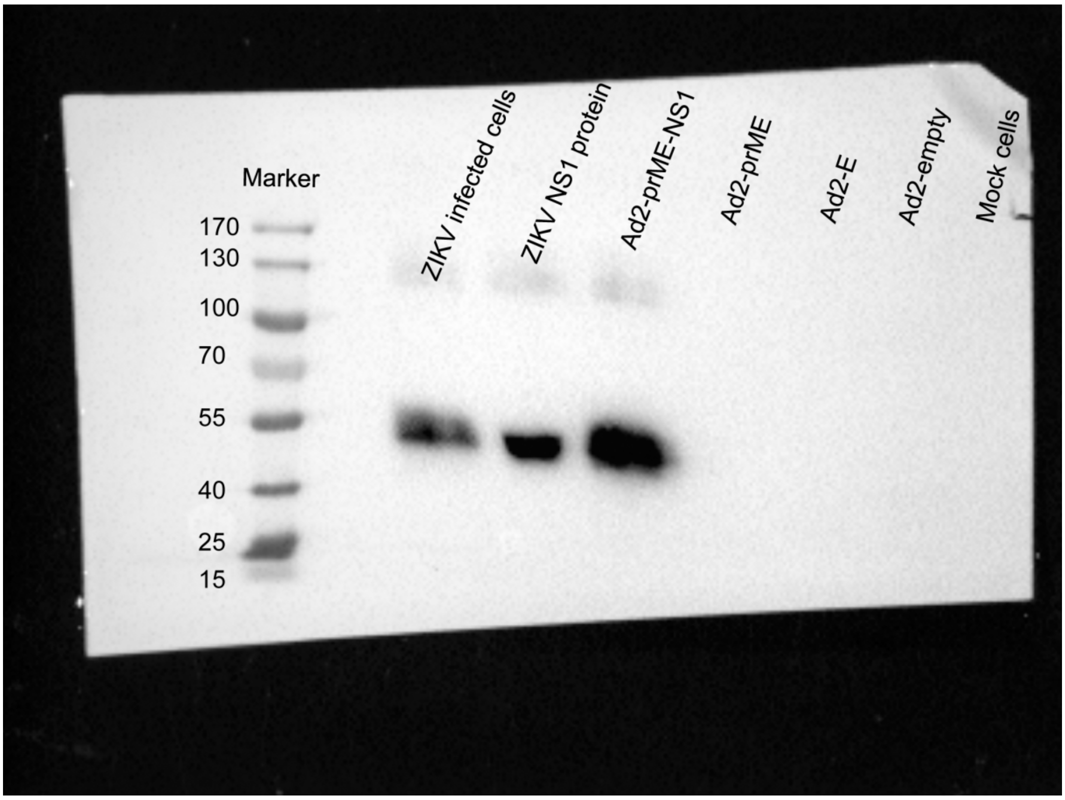

Supplementary Figure S13. NS1 proteins expressed by Ad2-prME-NS1 are secreted to culture mediums. The culture supernatants of Vero cells infected with Ad2-prME-NS1, Ad2-prME, Ad2-E and Ad2-empty were assessed using Western-blot analysis with anti-NS1 antibody. ZIKV infected cells and purified NS1 proteins were used as positive controls, and mock infected cells were used as negative controls. The graph was taken with ChemiDoc MP Imaging System and shown.

Supplementary Tables

Supplementary Table S1. The ELISA titers of E-specific IgG and NS1-specific IgG induced by immunization.

Groups	3 weeks post immunization		12 weeks post immunization	
	E-specific IgG[a]	NS1-specific IgG	E-specific IgG	NS1-specific IgG
Ad2-prME-NS1	2999 (2717 to 3310)[b]	17378 (16220 to 18618)	1047 (912 to 1201)	4977 (4783 to 5179)
Ad2-prME	6607 (5863 to 7445)	ND[c]	1327 (1211 to 1454)	ND
Ad2-E	877 (756 to 1017)	ND	553 (511 to 599)	ND
Ad2-empty	ND	ND	ND	ND

[a]The titers were calculated as the reciprocal of the last serum dilution yielding an O.D.450 value higher than the cutoff, which was defined as twice the absorbance value of the blank control wells.

[b]The 95% confidence interval (CI) values were calculated as the mean ±1.96SEM.

[c]ND, not detectable.

Supplementary Table S2. The iters of neutralizing antibodies and inhibitory antibodies induced by immunization.

Groups	3 weeks post immunization		12 weeks post immunization	
	Neutralizing antibody (MN50)[a]	Inhibitory antibody (IC50)[b]	Neutralizing antibody (MN50)	Inhibitory antibody (IC50)
Ad2-prME-NS1	1778 (1387 to 2280) [c]	4624 (3487 to 6131)	324 (287 to 365)	493 (360 to 674)
Ad2-prME	2754 (2382 to 3184)	1109 (733 to 1678)	414 (375 to 457)	146 (112 to 192)
Ad2-E	68 (50 to 92)	51 (39 to 66)	17 (15 to 20)	18 (14 to 23)
Ad2-empty	ND[d]	ND	ND	ND

[a]Neutralizing titers were from the neutralization assay in which the cells were infected with ZIKV in the presence of sera dilutions for two hours and then the infected cells were cultured in the absence of immune sera for another 4 days. MN50 titers were calculated as the reciprocal of the sera dilution at which the number of infected cells was reduced by 50%.

[b]Inhibitory titers were from the inhibition assay in which the immune sera were present in culture medium all the time, including after Vero cells were infected with ZIKV. IC50 titers were calculated as the reciprocal of the sera dilution at which the number of infected cells was reduced by 50%.

[c]The 95% confidence interval (CI) values were calculated as the mean ±1.96SEM.

[d]ND, not detectable.

Supplementary Table S3. The male and female neonates in each group included in the challenge assay.

Groups	born at 6 weeks post immunization		born at 15 weeks post immunization	
	Male	Female	Male	Female
Ad2-prME-NS1	6	3	5	4
Ad2-prME	5	7	4	4
Ad2-E	5	5	3	5
Ad2-Empty	5	5	4	7
Healthy control[a]	5	6	4	6

[a]Healthy control neonates received 20μl PBS via intraperitoneal injection.

Supplementary Materials and Methods

Viruses

The challenge strain of ZIKV was Zika virus/GZ02/2016 (GenBank KX056898.1), which was isolated from the urine sample of a ZIKV-infected patient returning from Venezuela to Guangzhou and propagated on Vero cells (CCL81; ATCC, Bethesda, MD, USA).[1] The viral stocks were titrated using plaque-forming assays on Vero cells and stored at -80 ºC.

Ad2 vectored ZIKV vaccines

The construction and production of recombinant Ad2 vectored ZIKV vaccines were performed according to previously described methods.[2] In brief, the coding sequence for ZIKV E protein, prM/M protein and NS1 protein (all derived from Zika virus isolate 1_0080_PF, GenBank ANO46313.1) was optimized according to mammalian codon usage and synthesized (Genscript, China). E-coding sequence was fused with a signal sequence at the N' terminal by overlap PCR and inserted into a shuttle vector pGA1 to obtain pGA1-E. The coding sequences for prM/M and E were fused with a signal sequence at the N' terminal by overlap PCR and inserted into pGA1 to obtain pGA1-prME. The sequence corresponding to prM/M-E was fused to NS1-coding sequence with the

addition of a self-cleaving 2A linker, and inserted into pGA1 to obtain pGA1-prME-NS1. Subsequently, pGA1-E, pGA1-prME, and pGA1-prME-NS1 were linearized by enzymatic digestion with BstZ17I and SgrAI (New England Biolabs, Ipswich, MA, USA), and subjected to homologous recombination with linearized pAd2ΔE1ΔE3 to obtain pAd2-E, pAd2-prME, and pAd2-prME-NS1, respectively. Finally, the genomes for Ad2-E, Ad2-prME, and Ad2-prME-NS1 were released by enzymatic digestion with PacI (New England Biolabs, Ipswich, MA, USA), transfected into HEK293 cells (CRL-N268, ATCC), and then rescued and propagated. Purified viral stocks were obtained by Cesium chloride (CsCl) gradient centrifugation and stored at -80ºC.

Western blot analysis

To analyze the expression of ZIKV E protein and NS1 protein mediated by Ad2 vectored ZIKV vaccines, Vero cells seeded on 60mm plates were infected by Ad2-E, Ad2-prME, Ad2-prME-NS1, and Ad2-empty at 100 viral particles (vp) per cell. At 48 h post infection, equal amount of the cell lysates and culture supernatants from each preparation were harvested separately and subjected to SDS-PAGE. Protein bands were transferred to polyvinylidene difluoride (PVDF) membranes, which were then blocked at room temperature (RT) for 1 h in PBST (PBS, pH7.4 with 0.05% Tween 20) with 5% of skim milk. Subsequently, the membranes

were incubated with a monoclonal anti-ZIKV E antibody (8D10, 0.5 μg/mL, unpublished data) or a monoclonal anti-ZIKV NS1 antibody B4 (1 μg/mL, Abcam, Cambridge, UK) at RT for 1 h. Finally, the membranes were incubated with horseradish peroxidase (HRP) conjugated goat anti-human IgG (Zsbio, China) at RT for 1 h and developed by Immobilon Western Chemiluminescent HRP Substrate (Millipore, Billerica, MA, USA). The images were taken using ChemiDoc MP Imaging System (Bio-Rad, Hercules, CA, USA), or by scanning the exposed films.

Negative-stain electron microscopy

Vero cells were infected by Ad2-E, Ad2-prME, Ad2-prME-NS1, or an empty control vector Ad2-empty at 100 vp per cell. At 48 h post infection, culture supernatants were harvested, condensed and fixed with 2.5% glutaraldehyde (Sigma-Aldrich, St. Louis, MO, USA) in 0.1 M sodium cacodylate buffer, pH 7.3, for 30 min. Then, 10 μl samples were dropped gently onto copper grids which were coated with Formvar film (Ted Pella, Redding, CA, USA), and remained for 2 min for the adherence of the condensed culture supernatants. The drop was gently dried with filter paper, followed by negative staining with 5% uranyl acetate for 30 sec, and then gently dried with filter paper. ZIKV was also examined similarly. The images were obtained using transmission electron microscope FEI

Tecnai Spirit (FEI Company, Hillsboro, OR, USA) operating at 120 kV.

Adult mouse ZIKV challenge model

To evaluate the protective efficacy of Ad2 vectored ZIKV vaccines in adult mice, six-week-old female BALB/c mice were immunized with each vaccine candidate at 1×10^{10} vp per mouse in 100 μl PBS through intramuscular injection. Mice immunized with Ad2-empty were used as negative controls. At three weeks after the first immunization, mice were boosted with the same dosage of the respective vaccine candidates. At three weeks after the booster immunization, the mice were intravenously challenged with 2.4×10^2 PFU ZIKV. The serum samples were harvested on days 1 and 4 post challenge and subjected to viral load analysis. To prepare convalescence sera (ZIKV sera), fifteen-week-old female BALB/c mice were infected with ZIKV at 1.2×10^5 PFU per mice through intraperitoneal injection. At 2 weeks post infection, mice were sacrificed and the serum samples were collected.

Depletion of E-binding and NS1-binding antibodies

Depletion of E-binding or NS1-binding antibodies was performed according to previously described methods with minor modifications.[3-6] In brief, 0.2 ml Ni-NTA Agarose beads (Qiagen, Germany) were attached with 50 μg purified ZIKV E-His protein or NS1-His protein or both (Sino

Biological, China) at 37°C for 2 h. The beads were then washed twice with PBS by centrifuging at 12000×g for 1 min. Subsequently, 0.2 ml Ad2-prME-NS1 immune sera or ZIKV sera were added with the labelled beads and incubated at 37°C for 2 h. Finally, the incubation mixture was centrifuged at 12000 × g for 5 min and the supernatants were harvested and subjected to ELISA and inhibition assay as mentioned above.

Enzyme-linked immunosorbent assay (ELISA)

In brief, 96-well Nunc MaxiSorp plates (Thermo Fisher Scientific, Waltham, MA, USA) were coated with 1 μg/ml purified ZIKV E or NS1 protein (Sino Biological) and incubated at 4ºC overnight. After blocking with blocking buffer for 1 h, the plates were washed with PBST. Serum samples from immunized adult mice or pups were serially diluted and added to the plates, and incubated at 37ºC for 2 h. The plates were then incubated with HRP-conjugated anti-mouse IgG (Zsbio) at RT for 1 h. Finally, the plates were developed using 3,3',5',5-Tetramethylbenzidine (TMB) HRP substrate (KPL, Gaithersburg, MD, USA), stopped with 1 M H2SO4 and the optical density was measured at 450 nm by Synergy™ HT Multi-Mode Microplate Reader (BioTek Instruments, Winooski, VT, USA). The cutoff values were defined as twice the mean absorbance value of the blank control wells.

FACS-based neutralization test (FNT)

The assay was performed according to a previously reported method with minor modification.[7] Serial dilutions of serum samples were mixed with 4×10^4 PFU ZIKV, incubated at 37°C for 1 hour and infected onto 2×10^4 Vero cells in 96-well flat-bottom plates. After incubation for 2 hours, cells were washed twice with PBS and the infection mixture were replaced with DMEM containing 2% FBS. Four days later, the cells were fixed and permeabilized using BD Cytofix/Ctyoperm™ (BD Biosciences, Bedford, MA, USA) according to the manufacturer's protocol, and stained with a monoclonal mouse anti-flavivirus antibody 4G2 (Millipore). The cells were then incubated with a PE-labelled goat anti-mouse IgG antibody (Biolegend, San Diego, CA, USA) and analyzed by Accuri C6 flow cytometry (BD Biosciences). The neutralization titer (microneutralization 50%, MN50) is calculated as the serum dilution at which the infection was reduced by 50% in comparison with virus-only control wells.

FACS-based inhibition test (FIT)

The assay was performed according to a previously reported method with minor modification.[8] Serial dilutions of serum samples were mixed with 4×10^4 PFU ZIKV, incubated at 37°C for 1 hour and infected onto 2×10^4 Vero cells in 96-well flat-bottom plates. Herein the infection mixtures

were not washed off so the antibodies were present in the culture media throughout the culture. Four days later, the cells were fixed and permeabilized using BD Cytofix/Ctyoperm™ (BD Biosciences) according to the manufacturer's protocol, and stained with a monoclonal mouse anti-flavivirus antibody 4G2 (Millipore). The cells were then incubated with a PE-labelled goat anti-mouse IgG antibody (Biolegend) and analyzed by Accuri C6 flow cytometry (BD Biosciences).. The half maximal inhibitory concentration titer (IC50) is calculated as the serum dilution at which the infection was reduced by 50% in comparison with virus-only control wells.

Neurological analysis

Fifteen days after challenge, the neurological symptoms of the neonates were scored in a blinded manner according to previously described methods.[9] In brief, for each hindlimb and forelimb, the neurological scores were designated as: 0, no signs; 1, weakness or altered gait; 2, paresis; 3, full paralysis. For the tail, the neurological scores were designated as: 0, no signs; 1, half paralysis; 2, full paralysis. The score of a neonatal mouse was calculated as the sum of the scores from each hindlimb and forelimb and the tail. Thus, a fully paralyzed animal would obtain a score of 14, whereas mortality equals a score of 15. Finally, the mice were euthanatized and the brain tissues and the testis were harvested

and subjected to viral load analysis.

Histology analysis

In brief, the neonatal brains were harvested at the time of autopsy and immediately fixed in 10% neutral buffered formalin for 7 days, and then transferred into 70% ethanol. Individual lobes of brain biopsy material were placed in processing cassettes, dehydrated through a serial alcohol gradient, and embedded in paraffin wax blocks. Before staining, 5-μm-thick tissue sections were dewaxed in xylene. The tissue sections were then incubated with hematoxylin solution for 15 min and rinsed in water. Subsequently, the slides were stained with Eosin solution for 5 min and washed. Finally, the slides were successively incubated with 70% ethanol for 20 sec, 90% ethanol for 20 sec, 100% ethanol for 1 min and xylene for 3 min. The images were pictured using a real-time microscopic image acquisition system Motic VM V1 Version 1.1 (Motic, China).

Real-time RT-PCR assay

The ZIKV viral loads in the neonatal brain and testis tissues and the plasma of ZIKV-infected adult mice were measured using Real-time RT-PCR as described previously.[10] In brief, total RNA was extracted from the brains of each pup using the RNeasy lipid tissue mini kit (Qiagen); total RNA was extracted from the testis of each male pup and from the

plasma of each mice using RNeasy Mini kit (Qiagen). Total RNA was subjected to one-step real-time RT-PCR using QuantiTect SYBR Green RT-PCR Kit (Qiagen) according to manufactory's protocol. The primer set includes the forward primer (NS5 F: 5′-TGGAGGCTGAGGAAGTTCTAG-3′), and reverse primer (NS5 F: 5′-CTTCACAACGCAATCATCTCCACTG -3′). The amplification procedures were set up as the following: initial denaturation at 95 °C for 10 min; 40 cycles at 95 °C for 30 s, 55 °C for 30 s, 72 °C for 30 s, and a melting curve was produced at 65 °C to 95 °C with an increment of 0.5 °C per cycle for 5 s. The standard curve was constructed with serial dilutions of ZIKV RNA fragments corresponding to the NS5 region generated by *in vitro* transcription. The lower detection limits were determined by serial dilutions of ZIKV RNA fragments in ZIKV-negative RNA extracts from brain or testis tissues or plasma of healthy control mice, similar to another study.[11] The detection limit for ZIKV viral RNA was about 1×10^4 copies per gram tissue or 1×10^2 copies per ml plasma. The viral loads were calculated as the ZIKV genome copies per gram tissue or per ml plasma.

Data process and statistics

Comparisons of the binding, neutralizing and inhibitory antibody titers among different immunization groups were conducted by one-way

ANOVA or Student’s t-test. Comparisons of the body weight, neurological symptoms scores and the viral loads among different challenge groups were also conducted by one-way ANOVA. All the statistical analyses were computed with SPSS version 13.0 (SPSS Inc., Chicago, IL, USA), and p values less than 0.05 were considered statistically significant. The data graphs were generated with GraphPad Prism version 7 (GraphPad Software, La Jolla, CA, USA). The illustrations were generated with Microsoft PowerPoint version 2010 (Microsoft, Redmond, NY, USA) and Photoshop version CS2 (Adobe Systems Incorporated, San Jose, CA, USA), and the figures were created with Photoshop version CS2 (Adobe Systems Incorporated).

Supplementary References

1. Zhang, F.C., Li, X.F., Deng, Y.Q., Tong, Y.G. & Qin, C.F. Excretion of infectious Zika virus in urine. *The Lancet. Infectious diseases* **16**, 641-642, (2016).
2. Zhang, Y., *et al.* Effects of the fusion design and immunization route on the immunogenicity of Ag85A-Mtb32 in adenoviral vectored tuberculosis vaccine. *Human vaccines & immunotherapeutics* **11**, 1803-1813, (2015).
3. Collins, M.H., *et al.* Lack of Durable Cross-Neutralizing Antibodies Against Zika Virus from Dengue Virus Infection. *Emerging infectious diseases* **23**, 773-781, (2017).
4. Chao, D.Y., Galula, J.U., Shen, W.F., Davis, B.S. & Chang, G.J. Nonstructural protein 1-specific immunoglobulin M and G antibody capture enzyme-linked immunosorbent assays in diagnosis of flaviviral infections in humans. *Journal of clinical microbiology* **53**, 557-566, (2015).
5. Patel, B., *et al.* Dissecting the human serum antibody response to secondary dengue virus infections. *PLoS neglected tropical diseases* **11**, e0005554, (2017).
6. Williams, K.L., Wahala, W.M., Orozco, S., de Silva, A.M. & Harris, E. Antibodies targeting dengue virus envelope domain III are not required for serotype-specific protection or prevention of enhancement in vivo. *Virology* **429**, 12-20, (2012).
7. Stettler, K., *et al.* Specificity, cross-reactivity, and function of antibodies elicited by Zika virus infection. *Science* **353**, 823-826, (2016).
8. Poungpair, O., *et al.* Generation of human single-chain variable fragment antibodies specific to dengue virus non-structural protein 1 that interfere with the virus infectious cycle. *mAbs* **6**, 474-482, (2014).
9. Weaver, A., *et al.* An elevated matrix metalloproteinase (MMP) in an animal model of multiple sclerosis is protective by affecting Th1/Th2 polarization. *Faseb Journal* **19**, 1668-1670 (2005).
10. Li, F., *et al.* AXL is not essential for Zika virus infection in the mouse brain. *Emerging microbes & infections* **6**, e16, (2017).
11. Ren, P., *et al.* Evaluation of Aptima Zika Virus Assay. *Journal of clinical microbiology* **55**, 2198-2203, (2017).
